# Transfer of Restrictive Episiotomy Practice with Low OASIS Rate from Tertiary to Primary Care Level—Lessons Learned and Future Challenges

**DOI:** 10.3390/healthcare14172714

**Published:** 2026-08-25

**Authors:** Ingrid Marton, Matija Prka, Ana Tikvica Luetić, Domagoj Dokozić, Dubravko Habek, Sanja Rogina Curman, Zlatko Hrgović

**Affiliations:** 1 University Department of Gynecology and Obstetrics, Clinical Hospital “Sveti Duh”, Sveti Duh 64, 10 000 Zagreb, Croatia; ingridmarton@gmail.com (I.M.); matija.prka@gmail.com (M.P.); ana.luetic@unicath.hr (A.T.L.); srogina@unicath.hr (S.R.C.); 2 School of Medicine, Faculty of Health Studies, Catholic University of Croatia, Ilica 244, 10 000 Zagreb, Croatia; dhabek@unicath.hr; 3 Department of Gynecology and Obstetrics, Požega General County Hospital, Osječka 107, 34 000 Požega, Croatia; domagoj_dokozic@yahoo.com; 4 University Department of Gynecology and Obstetrics, Clinical Hospital “Merkur”, Zajčeva 19, 10 000 Zagreb, Croatia; 5 School of Medicine, Johann Wolfgang Goethe University, 60629 Frankfurt am Main, Germany

**Keywords:** restrictive episiotomy, obstetric anal sphincter injuries, manual perineal protection, staff training

## Abstract

**Highlights:**

**What are the main findings?**

- Nowadays, it is necessary to find a balance (when using mediolateral or lateral episiotomy) between an optimal overall episiotomy rate of <30% (primiparas: <50%; multiparas: <10%) and a corresponding overall OASIS rate of up to 1%.
- Restrictive episiotomy was implemented at Clinical Hospital “Sveti Duh” (tertiary care level) in 2010 and at Požega General County Hospital (primary care level) in 2012.
- The implementation of restrictive episiotomy and programs aimed at maintaining an OASIS rate of <1% is not possible without the continuous education of both midwives and physicians. Some educational programs successfully transfer knowledge and training from the tertiary to the primary care level, including important interventions such as lateralization of episiotomy, slowing the expulsion of the fetal head, manual perineal protection (MPP), and performing episiotomy strictly according to medical indications.

**What are the implications of the main findings?**

- Specialists in obstetrics and gynecology currently working at Požega General County Hospital, who completed their training at Clinical Hospital “Sveti Duh”, between 2008 and 2010, implemented a set of obstetric measures in their clinical setting.
- These obstetric measures were: (1) performing episiotomy strictly according to medical indications; (2) individualized patient assessment; (3) patience during the second stage of labor; (4) critical judgment by both the obstetrician and the midwife, the availability of an experienced and skilled midwife, and the cooperation of the woman in labor; and (5) education of all birth attendants.
- The implementation of all these measures resulted in reductions in both episiotomy and OASIS rates at the two hospitals. However, our results clearly show that the discontinuation of staff training and organizational problems can be discouraging for staff members and may result in inappropriate clinical practice, which is not necessarily more likely to occur in small than in large clinical settings.

**Abstract:**

**Introduction:** For more than 40 years, obstetricians and midwives worldwide have continued to debate the significance of episiotomy and methods of reducing the rate of obstetric anal sphincter injuries (OASIS). **Methods:** A concise review of relevant recently published articles on obstetric perineal trauma was conducted, together with an analysis and comparison of the annual rates of episiotomy and OASIS (primary outcomes) at Clinical Hospital “Sveti Duh”, Zagreb (tertiary care level), and Požega General County Hospital, Požega (primary care level), over a ten-year period (2015–2024). **Results:** Routine (liberal) episiotomy has been abandoned in clinical practice worldwide, and restrictive (selective) episiotomy based on strict medical indications has gained widespread acceptance. Lateralization of episiotomy, slowing the expulsion of the fetal head, and manual perineal protection represent an important set of preventive interventions for avoiding OASIS. Restrictive episiotomy was implemented at Clinical Hospital “Sveti Duh” in 2010 and at Požega General County Hospital in 2012. A statistically significant difference in the episiotomy rate was observed between the two hospitals during the study period 2015–2019 (tertiary care level, 22.0% vs. primary care level, 17.5%), and an even greater difference was observed during 2020–2024 (tertiary care level, 31.0% vs. primary care level, 17.0%). At the tertiary care level, we detected a slight increase in the OASIS rate during 2020–2024 compared with 2015–2019, whereas a slight decrease was observed at the primary care level. No statistically significant difference in OASIS rates was detected between the two hospitals or between the two study periods, indicating that the OASIS rate remained stable at below 1%. **Discussion:** Nowadays, it is necessary to find a balance (when using mediolateral or lateral episiotomy) between an optimal overall episiotomy rate of <30% (primiparas: <50%; multiparas: <10%) and a corresponding overall OASIS rate of up to 1%. Several measures were important for the successful restrictive approach to episiotomy at Clinical Hospital “Sveti Duh” and, subsequently, at Požega General County Hospital: (1) performing episiotomy strictly according to medical indications; (2) individualized patient assessment; (3) patience during the second stage of labor; (4) critical judgment by both the obstetrician and the midwife, the availability of an experienced and skilled midwife, and the cooperation of the woman in labor; and (5) education of all birth attendants, including the transfer of knowledge and training from the tertiary to the primary care level. The implementation of all these measures resulted in reductions in both episiotomy and OASIS rates at the two hospitals. However, our results clearly show that the discontinuation of staff training and organizational problems can be discouraging for staff members and may result in inappropriate clinical practice, which is not necessarily more likely to occur in small than in large clinical settings.

## 1. Introduction

Episiotomy is the surgical enlargement of the vaginal orifice through an incision in the perineum during the final part of the second stage of labor or delivery [1]. It is the second most common surgical procedure worldwide, after clamping and cutting the umbilical cord [2]. Episiotomy must be defined by the location of the incision’s origin, its direction and length, and the timing of the incision. Seven types of episiotomy have been identified [1], but only three (midline, mediolateral, and lateral) are routinely performed. It is clearly indicated in cases of fetal compromise and, by consensus, in instrumental deliveries.

Current clinical guidelines and systematic reviews recommend avoiding routine (liberal) episiotomy [3], as no benefit of this practice has been demonstrated [4]. Traditionally, episiotomy has been a routine component of instrumental delivery, with the primary aim of preventing obstetric anal sphincter injuries (OASIS). Midline episiotomy is associated with an increased risk of OASIS [3] and consequent functional impairment, including fecal incontinence [4]. Evidence has shown that mediolateral episiotomy reduces the risk of OASIS in instrumental deliveries [5,6]. Routine use of mediolateral episiotomy in instrumental delivery is even recommended by the National Institute for Health and Care Excellence (NICE) [7].

Carroli and Belizán [8] established that a restrictive episiotomy rate above 30% is not clinically justified. Routine episiotomy clearly does not reduce perineal or vaginal trauma, and selective episiotomy should be performed in women for whom instrumental delivery is not intended, according to the Cochrane meta-analysis [9]. When using restrictive episiotomy and different episiotomy types (mediolateral and lateral), it is necessary to achieve a balance between an optimal overall episiotomy rate of <30% (primiparas: <50%; multiparas: <10%) and maintaining a low OASIS rate of up to 1% [10].

Like all surgical procedures, episiotomy has both short-term complications (hematoma, dehiscence, and infection) and long-term complications (perineal pain and sexual dysfunction) [1]. OASIS is recognized as one of the main causes of pelvic floor dysfunction (PFD) during the postpartum period. It can have a significant impact on quality of life, especially because of long-term complications such as rectovaginal fistula and anal incontinence. Therefore, various obstetric measures and strategies have been implemented to reduce the OASIS rate, including lateralization of episiotomy and manual perineal protection (MPP).

According to the guidelines of the Royal College of Obstetricians and Gynaecologists (RCOG), an acceptable OASIS rate is ≤1% of all vaginal deliveries [11]. This can be achieved by introducing interventions such as lateralization of episiotomy, slowing the expulsion of the fetal head, MPP, and performing episiotomy strictly according to medical indications [10].

MPP is an obstetric intervention performed at the very end of the second stage of labor and has been used in clinical practice for a long time. Its purpose is to mitigate the risk of perineal trauma and its consequences for PFD. Three MPP techniques have been introduced into clinical practice: (1) hands-on: perineal protection during crowning of the fetal head when the perineum is stretched; (2) hands-poised: perineal protection only in the event of an imminent (severe) perineal tear; and (3) hands-off: no perineal protection during labor [10]. Nowadays, we distinguish between two variants of the hands-on technique: the Finnish and Viennese methods, which have minor but distinct differences [10]. Many studies, including that by Naidu et al. [12], have investigated and demonstrated that perineal support reduces the OASIS rate, although they did not specify the exact technique used.

According to a recent study by Laine et al. [13], the OASIS rate can be successfully reduced after adjustment for the individual’s age and country of birth, infant birthweight, use of epidural analgesia, and episiotomy. The decrease in OASIS incidence among nulliparous individuals with singleton spontaneous vaginal births can be attributed to the widespread adoption of MPP techniques following comprehensive staff training [13]. In a recently published study by Laine and Räisänen [14], the increased incidence of OASIS in vaginal birth after cesarean delivery (VBAC) was explained by a higher prevalence of recognized OASIS risk factors, such as operative vaginal delivery and neonatal birthweight above 4000 g. The findings are generalizable to obstetric settings with similar clinical practices, including the use of mediolateral or lateral episiotomy, vacuum and forceps delivery, and structured MPP during the second stage of labor.

A restrictive approach to episiotomy was introduced at Clinical Hospital “Sveti Duh” (tertiary care level) in 2010, when the episiotomy rate fell below 30% for the first time [15]. The same approach was introduced at Požega General County Hospital (primary care level) a few years later, in 2012 [16]. In both settings, restrictive lateral episiotomy and MPP have been implemented in everyday clinical practice. Specialists in obstetrics and gynecology currently working at Požega General County Hospital who completed their training at Clinical Hospital “Sveti Duh” between 2008 and 2010 implemented both practices in their clinical setting. The implementation of restrictive episiotomy and programs aimed at maintaining an OASIS rate of <1% is not possible without continuous education of both midwives and physicians. Some educational programs successfully transfer knowledge and training from the tertiary to the primary care level.

The aim of this study was to compare the rates of episiotomy, OASIS, and cesarean section (CS) between two hospitals, with particular attention to trends in these rates within each hospital and differences between the hospitals. Understanding trends within each clinical setting provides a clear picture of developments in each obstetric team with regard to education, knowledge transfer, and practical skills. Critical analysis is crucial for improving everyday clinical practice.

## 2. Materials and Methods

This article presents a retrospective analysis of two different hospitals (primary and tertiary perinatal centers) that share very similar clinical practices, particularly regarding MPP and indications for episiotomy. Although women giving birth in the tertiary obstetric setting had high-risk pregnancies, we decided to analyze and compare how changes in training programs can improve or even impair clinical practice.

The study was conducted at two perinatal centers between 2015 and 2024. No specific inclusion or exclusion criteria were applied because of its retrospective design. We calculated the annual total number of deliveries and the number of vaginal deliveries and analyzed and compared annual rates of episiotomy and OASIS (primary outcomes) and cesarean section (CS; secondary outcome) across two study periods (2015–2019 vs. 2020–2024) at a tertiary perinatal center (Clinical Hospital “Sveti Duh”, Zagreb) and a primary perinatal center (Požega General County Hospital, Požega).

We collected data from electronic medical records that are official and publicly available according to our healthcare provider. Data are collected annually for evaluation by the healthcare provider and the Ministry of Health. The analyzed data were extracted by an obstetrician working at the primary center and a midwife working at the tertiary perinatal center.

At both centers, left lateral episiotomy with an incision angle of approximately 60° and MPP are traditionally performed. Both centers have a long history of MPP, with the Viennese method performed at each delivery. In accordance with national recommendations and in an attempt to standardize clinical practice, lateral episiotomy is strongly recommended for all instrumental deliveries [17]. Indications for episiotomy were similar at both centers: fetal hypoxia requiring expedited delivery, instrumental delivery (vacuum extraction), fetal macrosomia, shoulder dystocia, a prolonged second stage of labor, and a short perineum.

At both centers, rectal examination or palpation is performed immediately after delivery in women with a second-degree perineal tear and/or episiotomy. The degree of perineal tear is diagnosed according to the Sultan classification [10]. At both centers, a senior obstetrician makes the diagnosis and performs the surgical intervention. All cases of OASIS are recorded in both hospital and national registries.

All data was collected in the Microsoft Excel sheets and summarized with percentages. MedCalc^®^ Statistical Software version 23.6.1 (MedCalc Software Ltd., Ostend, Belgium; https://www.medcalc.org; 2026) was used to compare rates between institutions and time periods and obtain Poisson 95% confidence intervals and *p* values. *p* values less than 0.05 were considered significant.

## 3. Results

Restrictive episiotomy was implemented at Clinical Hospital “Sveti Duh”, Zagreb, in 2010 and at Požega General County Hospital in 2012.

Between 2015 and 2024, the number of deliveries differed significantly between the tertiary perinatal center (approximately 2500–3500 per year) and the primary perinatal center (approximately 350–500 deliveries per year) (Table 1 and Table 2).

During the study period 2015–2019, the total number of deliveries at Clinical Hospital “Sveti Duh” was 13,607, including 10,613 vaginal deliveries (78.0%) and 2994 cesarean sections (22.0%). The total number of episiotomies during the study period was 2333 (22.0%), and the OASIS rate was 0.50% (Table 1). During 2020–2024, the total number of deliveries at the tertiary center was 15,674, including 11,862 vaginal deliveries (75.7%) and 3812 cesarean sections (24.3%). The total number of episiotomies during this period was 3691 (31.1%), and the OASIS rate was 0.69% (Table 1). At the tertiary center during 2015–2019, the lowest episiotomy rate, 18.6%, was recorded in 2018, with an OASIS rate of 0.38%. The lowest OASIS rate, 0.28%, was recorded in 2017, with an episiotomy rate of 20.7%. At the same center during 2020–2024, the lowest episiotomy rate, 26.2%, was recorded in 2020, with an OASIS rate of 0.93%. The lowest OASIS rate, 0.52%, was recorded in 2023, with an episiotomy rate of 31.7% (Table 1). From 2020 onward, the episiotomy rate increased continuously. In 2021, it exceeded 30%, and in 2024, it reached 35%. The OASIS rate was relatively stable during 2015–2019 but unexpectedly began to increase alongside the episiotomy rate from 2020 onward.

During the study period 2015–2019, the total number of deliveries at Požega General County Hospital was 2278, including 1754 vaginal deliveries (77.0%) and 524 cesarean sections (23.0%). The total number of episiotomies during this period was 307 (17.5%), and the OASIS rate was 0.46% (Table 2). During 2020–2024, the total number of deliveries at Požega General County Hospital was 1930, including 1350 vaginal deliveries (69.9%) and 580 cesarean sections (30.1%). The total number of episiotomies during this period was 230 (17.0%), and the OASIS rate was 0.37% (Table 2). At Požega General County Hospital during 2015–2019, the lowest episiotomy rate, 11.7%, was observed in 2016, with an OASIS rate of 0.56%. The lowest OASIS rate, 0%, was recorded in 2019, when the episiotomy rate was highest, at 23.0%.

At the same center during 2020–2024, the lowest episiotomy rate, 11.4%, was recorded in 2023, with an OASIS rate of 0.0% (Table 2). In 2015, the annual episiotomy rate was very low, at 11.7%, but it subsequently increased and reached 23.0% in 2019. From 2020 to 2024, the episiotomy rate decreased markedly and reached 11.4% in 2023. The OASIS rate was stable and below 0.6% from 2015 to 2018 but increased to 0.72% in 2021. From 2021 onward, the OASIS rate decreased, reaching 0% in 2023 and 2024. In 2019, we observed the highest episiotomy rate (23%) and the lowest OASIS rate (0%). Interestingly, when the episiotomy rate was lowest (11.4%), the OASIS rate was approximately 0% (Table 2).

At the tertiary perinatal center during 2015–2019, the vaginal delivery rate was 78.0% and the CS rate was 22.0%. The episiotomy rate (22.0%) and OASIS rate (0.50%) were low and acceptable. Similar results were obtained at the primary perinatal center. During 2020–2024, at the tertiary center, we observed an increase in the number of deliveries, a slight decrease in the vaginal delivery rate (75.7%), and an increase in the CS rate (24.3%), accompanied by a significant increase in the episiotomy rate (31.0%) and an increase in the OASIS rate (0.69%). At the same time, at the primary center, we observed a decrease in the vaginal delivery rate (69.9%), a significant increase in the CS rate (30.1%), a stable episiotomy rate (17.0%), and a slight decrease in the OASIS rate (0.37%). At both hospitals, we unfortunately observed a statistically significant increase in the CS rate during 2020–2024 (Table 3).

We observed a statistically significant difference in episiotomy rates during the two study periods. A difference in episiotomy rates was observed between the two hospitals during 2015–2019 (tertiary center, 22.0% vs. primary center, 17.5%), and an even greater difference was observed during 2020–2024 (tertiary center, 31.0% vs. primary center, 17.0%).

At the tertiary center, we detected a slight increase in the OASIS rate during 2020–2024 compared with 2015–2019, whereas a slight decrease was observed at the primary center. 

However, OASIS rates were below 1% in each case, with no statistically significant differences in rates between the two centers or between the two study periods.

## 4. Discussion

The tertiary perinatal center at Clinical Hospital “Sveti Duh” was the first maternity unit in Croatia to introduce the concept of restrictive episiotomy, in 2010 [15]. Alongside restrictive episiotomy, a critical approach to reducing the OASIS rate was introduced. The incorporation of this concept into everyday clinical practice resulted in changes in obstetric practice, as reported in the first national survey on episiotomy [18]. From that time, and particularly during 2015–2019, a team of senior staff responsible for this obstetric improvement conducted training programs and workshops for midwives and obstetricians to improve their clinical skills in MPP, episiotomy, and OASIS.

MPP is an obstetric intervention performed at the very end of the second stage of labor and has been used in clinical practice for a long time. Its purpose is to mitigate the risk of perineal trauma and its consequences for PFD. Using a novel biomechanical model of the perineum, Jansova et al. [19] showed that the highest strain or tension throughout the perineal body during vaginal delivery was reduced by 30–39% with the hands-on technique compared with the hands-off technique. Moreover, the exact placement of the fingertips (thumb and index finger) on the perineal skin, together with their coordinated movement, plays an important role in reducing perineal tension [12]. Although some evidence suggests that the hands-poised technique is a safe and recommended approach to perineal management [20], a multidisciplinary group of experts in the United Kingdom reached a consensus that the hands-on technique should be recommended for MPP to prevent OASIS [21].

Nowadays, we distinguish between two variants of the hands-on technique: the Finnish and Viennese methods, which have minor but distinct differences [10]. In both techniques, the nondominant hand controls the speed of fetal head expulsion and facilitates extension of the fetal head. In the Finnish method, the clinician’s thumb and index or middle finger are used to support the posterior perineum while the other hand controls delivery of the head. The Viennese method is similar to the Finnish method but uses a distinct, specific application of pressure focused on reducing transverse perineal tension.

Many studies, including that by Naidu et al. [12], have investigated and demonstrated that perineal support reduces the OASIS rate, although they did not specify the exact technique used. Therefore, the work of Kleprlikova et al. is of great importance [22]. They conducted a survey in three maternity units in three countries (the Czech Republic, Slovenia, and the United Kingdom) that use MPP in everyday clinical practice. The results showed that although participants were aware of MPP, only a minority were familiar with the concept, indicating that it should be better understood and more widely accepted [22].

Senior obstetricians from Požega General County Hospital who completed their training programs at Clinical Hospital “Sveti Duh” between 2008 and 2010 implemented a set of established obstetric measures in their clinical setting. These measures were: (1) performing episiotomy strictly according to medical indications; (2) individualized patient assessment; (3) patience during the second stage of labor; (4) critical judgment by both the obstetrician and the midwife, the availability of an experienced and skilled midwife, and the cooperation of the woman in labor; and (5) education of all birth attendants. The implementation of all these measures resulted in reductions in both episiotomy and OASIS rates at the two perinatal centers [15,16].

Since 2015, some authors of this study (I.M., M.P., A.T.L., and D.H.) have conducted national training programs for physicians and midwives on MPP, episiotomy, and OASIS. Participants come from throughout Croatia and Southeastern Europe. Training in MPP is conducted using mannequin models, training in OASIS using biological tissue models (pig rectums), and training in episiotomy using bovine hearts.

Majority of obstetricians and midwives from both perinatal centers attended previously mentioned national training programs during the study period.

Many recommendations support restrictive rather than routine episiotomy; however, in assisted vaginal births, mediolateral or lateral episiotomy is strongly recommended because it has been shown to significantly reduce the incidence of OASIS [23]. Perrin et al. [24] clearly showed that episiotomy appears to protect against OASIS in nulliparous women at term only in operative vaginal deliveries. According to national guidelines, lateral episiotomy is mandatory for instrumental delivery (vacuum extraction) at both centers [17].

Moreover, our results are similar to those of Holowko et al. [25], who found no significant association between obstetric volume (i.e., the level of the perinatal center) and the OASIS rate, which is consistent with our findings.

From 2020 onward, we encountered several organizational problems at the tertiary perinatal center: (1) the departure of qualified midwives to other institutions or their retirement; (2) a generation gap among midwives; (3) staff resistance to obstetric skills training (MPP, episiotomy, and OASIS detection); and (4) a lack of interest and enthusiasm for the topic among both obstetricians and midwives. Together, these factors resulted in a significant increase in the episiotomy rate to >30%. These results are very concerning and far from those achieved in 2011–2012, when the episiotomy rate at our tertiary center was <20% [15,18].

We are aware of some limitations of our study. First of all, its retrospective design and consequent impact on: (1) differences in patient characteristics, (2) potential confounding between the two perinatal centers, and (3) interpretation of the study findings. Due to higher prevalence of high-risk pregnancies at the tertiary perinatal center, our primary goals (episiotomy and OASIS rates) could be affected. Regarding causal interpretations between staff training and changes in clinical practice, lack of direct assessment of exposure to and adherence to the training programs in both centers is present.

It is clear that Good Clinical Practice (GCP) cannot be implemented without continuous staff training and improvements in the organizational setting.

## 5. Conclusions

This research is a retrospective analysis of two different hospitals (primary and tertiary perinatal centers) that share very similar clinical practices, particularly regarding MPP and indications for episiotomy. In this manuscript, we analyzed and compared how changes in training programs can improve or even impair everyday clinical practice. This study highlighted the need for continuous training programs for both midwives and obstetricians. The main goal of these training programs should be the continuous improvement of GCP, which has substantial short- and long-term effects on the pelvic floor.

## Figures and Tables

**Table 1:** Number of deliveries (total), vaginal deliveries, cesarean sections, episiotomies and OASIS (with associated rates in brackets) at Clinical Hospital Sveti Duh.

Year	2015	2016	2017	2018	2019	2015–2019	2020	2021	2022	2023	2024	2020–2024	2015–2024
Total number of deliveries—N	2795	2762	2723	2746	2581	13,607	3057	3321	2977	3456	2863	15,674	29,281
Vaginal delivery—n (%)	2206 (78.9)	2190 (79.3)	2116 (77.7)	2119 (77.2)	1982 (76.8)	10,613 (78.0)	2378 (77.8)	2611 (78.6)	2279 (76.6)	2491 (72.1)	2103 (73.5)	11,862 (75.7)	22,475 (76.8)
Cesarean section—n (%)	589 (21.1)	572 (20.7)	607 (22.3)	627 (22.8)	599 (23.2)	2994 (22.0)	679 (22.2)	710 (21.4)	698 (23.4)	965 (27.9)	760 (26.5)	3812 (24.3)	6806 (23.2)
Episiotomy—n (%)	549 (24.9)	547 (25.0)	438 (20.7)	394 (18.6)	405 (20.4)	2333 (22.0)	624 (26.2)	801 (30.7)	739 (32.4)	790 (31.7)	737 (35.0)	3691 (31.1)	6024 (26.8)
OASIS—n (%)	11 (0.50)	12 (0.55)	6 (0.28)	8 (0.38)	16 (0.81)	53 (0.50)	22 (0.93)	18 (0.69)	14 (0.61)	13 (0.52)	15 (0.71)	82 (0.69)	135 (0.60)

**Table 2:** Number of deliveries (total), vaginal deliveries, cesarean sections, episiotomies and OASIS (with associated rates in brackets) at Požega General County Hospital.

Year	2015	2016	2017	2018	2019	2015–2019	2020	2021	2022	2023	2024	2020–2024	2015–2024
Total number of deliveries—N	478	453	453	449	445	2278	444	407	360	346	373	1930	4208
Vaginal delivery—n (%)	381 (79.7)	360 (79.5)	345 (76.2)	342 (76.2)	326 (73.3)	1754 (77.0)	316 (71.2)	277 (68.1)	262 (72.8)	245 (70.8)	250 (67.0)	1350 (69.9)	3104 (73.8)
Cesarean section—n (%)	97 (20.3)	93 (20.5)	108 (23.8)	107 (23.8)	119 (26.7)	524 (23.0)	128 (28.8)	130 (31.9)	98 (27.2)	101 (29.2)	123 (33.0)	580 (30.1)	1104 (26.2)
Episiotomy—n (%)	48 (12.6)	42 (11.7)	70 (20.3)	72 (21.1)	75 (23.0)	307 (17.5)	57 (18.0)	39 (14.1)	59 (22.5)	28 (11.4)	47 (18.8)	230 (17.0)	537 (17.3)
OASIS—n (%)	2 (0.52)	2 (0.56)	2 (0.58)	2 (0.58)	0	8 (0.46)	2 (0.63)	2 (0.72)	1 (0.38)	0	0	5 (0.37)	13 (0.42)

**Table 3:** Comparison of the two study periods (2015–2019 vs. 2020–2024) at University Hospital Sveti Duh Clinical and Požega General County Hospital regarding number of cesarean sections, episiotomies and OASIS (with associated rates in brackets).

	Clinical Hospital Sveti Duh			Požega General County Hospital			Inter Hospital Difference	
Study Periods	2015–2019	2020–2024	2015–2019 vs. 2020–2024	2015–2019	2020–2024	2015–2019 vs. 2020–2024	2015–2019	2020–2024
Cesarean section—n (%)	2994 (22.0)	3812 (24.3)	+2.3% (95%CI 1.2–3.4) *p* < 0.0001	524 (23.0)	580 (30.1)	+7.1% (95%CI 3.9–10.2) *p* < 0.0001	+1.0% (95%CI −1.1–+3.1) *p* = 0.3483	+5.7% (95%CI 3.4–8.1) *p* < 0.0001
Episiotomy—n (%)	2333 (22.0)	3691 (31.1)	+9.1% (95%CI 7.8–10.5) *p* < 0.0001	307 (17.5)	230 (17.0)	−0.4% (95%CI −3.4–+2.5) *p* = 0.757	−4.5% (95%CI −6.8–−2.2) *p* = 0.0002	−14.1% (95%CI −17.2–−11.0) *p* < 0.0001
OASIS—n (%)	53 (0.50)	82 (0.69)	+0.2% (95%CI −0.01–+0.3) *p* = 0.0639	8 (0.46)	5 (0.37)	−0.08% (95%CI −0.5–+0.3) *p* = 0.7145	−0.04% (95%CI −0.4–+0.3) *p* = 0.8110	−0.3% (95%CI −0.7–+0.1) *p* = 0.1686

## Data Availability

No new data were created or analyzed in this study. Data sharing is not applicable to this article.

## Abbreviations

The following abbreviations are used in this manuscript:

OASIS	Obstetric anal sphincter injuries
MPP	Manual perineal protection
CS	Cesarean section
NICE	National Institute for Health and Care Excellence
RCOG	Royal College of Obstetricians and Gynecologists
GCP	Good Clinical Practice
